# Body Composition Is a Predictor for Postoperative Complications After Gastrectomy for Gastric Cancer: a Prospective Side Study of the LOGICA Trial

**DOI:** 10.1007/s11605-022-05321-0

**Published:** 2022-04-29

**Authors:** Thaís T. T. Tweed, Arjen van der Veen, Stan Tummers, David P. J. van Dijk, Misha D. P. Luyer, Jelle P. Ruurda, Richard van Hillegersberg, Jan H. M. B. Stoot, LOGICA study group, Juul J. W. Tegels, Karel W. E. Hulsewe, Hylke J. F. Brenkman, Maarten F. J. Seesing, Grard A. P. Nieuwenhuijzen, Jeroen E. H. Ponten, Bas P. L. Wijnhoven, Sjoerd M. Lagarde, Wobbe O. de Steur, Henk H. Hartgrink, Ewout A. Kouwenhoven, Marc J. van Det, Eelco B. Wassenaar, Edwin S. van der Zaag, Werner A. Draaisma, Ivo A. M. J. Broeders, Suzanne S. Gisbertz, Mark I. van Berge Henegouwen, Hanneke W. M. van Laarhoven

**Affiliations:** 1 Department of Surgery, Zuyderland Medical Center, Heerlen and Sittard-Geleen, the Netherlands; 2 Department of Surgery, University Medical Center Utrecht, Utrecht University, Utrecht, the Netherlands; 3 Department of Surgery, Catharina Hospital, Eindhoven, the Netherlands

**Keywords:** Body composition, Skeletal muscle mass, Radiation attenuation, Gastrectomy, Chemotherapy

## Abstract

**Purpose:**

There is a lack of prospective studies evaluating the effects of body composition on postoperative complications after gastrectomy in a Western population with predominantly advanced gastric cancer.

**Methods:**

This is a prospective side study of the LOGICA trial, a multicenter randomized trial on laparoscopic versus open gastrectomy for gastric cancer. Trial patients who received preoperative chemotherapy followed by gastrectomy with an available preoperative restaging abdominal computed tomography (CT) scan were included. The CT scan was used to calculate the mass (M) and radiation attenuation (RA) of skeletal muscle (SM), visceral adipose tissue (VAT), and subcutaneous adipose tissue (SAT). These variables were expressed as *Z*-scores, depicting how many standard deviations each patient’s CT value differs from the sex-specific study sample mean. Primary outcome was the association of each *Z*-score with the occurrence of a major postoperative complication (Clavien-Dindo grade ≥ 3b).

**Results:**

From 2015 to 2018, a total of 112 patients were included. A major postoperative complication occurred in 9 patients (8%). A high SM-M *Z*-score was associated with a lower risk of major postoperative complications (RR 0.47, 95% CI 0.28–0.78, *p* = 0.004). Furthermore, high VAT-RA *Z*-scores and SAT-RA *Z*-scores were associated with a higher risk of major postoperative complications (RR 2.82, 95% CI 1.52–5.23, *p* = 0.001 and RR 1.95, 95% CI 1.14–3.34, *p* = 0.015, respectively). VAT-M, SAT-M, and SM-RA *Z*-scores showed no significant associations.

**Conclusion:**

Preoperative low skeletal muscle mass and high visceral and subcutaneous adipose tissue radiation attenuation (indicating fat depleted of triglycerides) were associated with a higher risk of developing a major postoperative complication in patients treated with preoperative chemotherapy followed by gastrectomy.

**Supplementary Information:**

The online version contains supplementary material available at 10.1007/s11605-022-05321-0.

## Introduction

Gastric cancer is the sixth most prevalent cancer and the third most common cause of cancer-related death worldwide.^1^ Perioperative chemotherapy followed by gastrectomy is the treatment of choice in the Western population.^2^ Approximately, 42% of all gastric cancer patients who undergo surgical resection develop a postoperative complication and 21% a major postoperative complication (Clavien-Dindo grade III or higher).^3,4^

Clinically, accurate prediction of major postoperative complication may help in the choice to refrain from surgery in very fragile patients or to improve the patient’s health status preoperatively. Several risk factors for a higher risk of postoperative complications and mortality have been identified (age, malnutrition, anemia, smoking, total gastrectomy). Yet, these factors do not fully explain the observed wide variation in postoperative complications after gastrectomy.^5,6^

Recently, sarcopenia, and other body composition parameters such as myosteatosis (lipid infiltration in skeletal muscle) have been identified as independent risk factors for postoperative complications.^7–9^ Sarcopenia is defined as a progressive loss of skeletal muscle strength in the presence of low skeletal muscle mass or skeletal muscle quality.^10–15^ An example of reduced muscle quality is myosteatosis which is associated with reduced physical fitness.^16^ For both lower and upper gastrointestinal surgery, previous studies have demonstrated that sarcopenia, myosteatosis, and other body composition parameters are associated with a worse postoperative outcome.^13,17–19^ For gastric cancer surgery, a recent meta-analysis including mostly Eastern studies showed that the odds of developing major postoperative complications and overall mortality were higher in patients with a low muscle mass.^9^ However, most the studies included in this meta-analysis were retrospective and used a wide variety of sarcopenia cut-off points. Furthermore, Western and Eastern gastric cancer population have important differences, impeding generalizability of Eastern studies on the Western population.^20^ Hence, there is a need for more prospective Western studies.

The aim of the current study was to evaluate body composition as predictor for postoperative complications in patients with gastric cancer treated with preoperative chemotherapy and gastrectomy.

## Materials and Methods

### Study Design

This is a multicenter, prospective, observational cohort side study of patients included in the Laparoscopic versus open gastrectomy for gastric cancer (LOGICA) trial.^21^ The current side study was initiated in 2015 together with the LOGICA trial. The LOGICA trial evaluated surgical and oncological outcomes between laparoscopic and open gastric surgery for gastric cancer. The results of the main trial were previously published.^21^ The current side study was conducted in compliance with the Dutch law and in accordance with the principles of the declaration of Helsinki. Written informed consent was obtained from all participating patients for inclusion in the LOGICA trial. The abdominal computed tomography (CT) scans of all LOGICA trial participants were pseudonymized and used for body composition analysis, as was approved by the Dutch Ethical Committee of Utrecht (medisch-ethische toetsingscommissie).

### Procedures

Clinical staging included gastroesophagoscopy with biopsy and a CT scan of the thorax and abdomen. All patients were discussed in a multidisciplinary tumor board meeting prior to treatment. Perioperative chemotherapy was recommended in all eligible patients with advanced tumors (cT3-4N0-3 or cT1-2N1-3). For each individual patient who underwent preoperative chemotherapy, the multidisciplinary tumor board of each individual hospital determined whether a restaging CT scan was made during the last courses of chemotherapy or after completion of chemotherapy. A restaging CT scan was thus not obligatory, as is in line with standard of care in the Netherlands.

In the LOGICA trial, patients were randomized in a 1:1 ratio between laparoscopic and open surgery.^21^ Surgical procedures included total or distal gastrectomy with total omentectomy and D2 lymphadenectomy, as previously described.^21^ Postoperative treatment protocols were in accordance with to the guidelines for Enhanced Recovery After Surgery (ERAS).^22^ Multiple quality control measures were included in the LOGICA trial, as previously described.^21^

### Patients and Data Collection

Patients included in the LOGICA trial were eligible for this study and therefore met the same inclusion criteria set for the trial.^21^ Both study arms (laparoscopic and open gastrectomy) were included. The primary analysis included the patients who underwent preoperative chemotherapy followed by a D2 gastrectomy. As this was an observational prospective side study, a restaging CT scan was not obligatory, and only patients with a restaging CT scan were included. Subgroup analyses were performed in patients who underwent primary surgery, by using the CT scan closest to the operation date (but within 6 months prior to the operation data). The distinction between the primary surgery group and preoperative chemotherapy group was made, since the primary surgery group was expected to consist of a more heterogeneous cohort of patients in worse clinical condition and with different preoperative body compositions, compared to the preoperative chemotherapy group.

For the purpose of the current prospective side study, the patients included in the LOGICA trial completed the Short Nutritional Assessment Questionnaire (SNAQ)^23^ and Groningen Frailty Index (GFI) questionnaire^24^ 1 week prior to gastrectomy. Higher questionnaire scores indicate more malnutrition or more frailty, respectively.

### Body Composition Analysis

For each abdominal CT scan, a single transverse slice at the level of the third lumbar vertebra (L3) was extracted by a single researcher trained in body composition analysis (TT). Total cross-sectional surface area (cm^2^) measurements of skeletal muscle tissue (SM), visceral adipose tissue (VAT), and subcutaneous adipose tissue (SAT) were performed using Slice-O-Matic 5.0® software using predefined Hounsfield unit (HU) ranges (− 29 to 150 HU, − 150 to − 50 HU, and − 190 to − 30 HU, respectively).^13,25,26^ Total cross-sectional surface area (cm^2^) of SM, VAT, and SAT was corrected for patient height to calculate the L3-index (cm^2^/m^2^). This parameter will be referred to as the mass (M) of these 3 tissues: SM-M, VAT-M, and SAT-M (Table 1).

**Table 1:** Variables and abbreviations

Variable	Abbreviation
**Skeletal muscle**	SM
**Visceral adipose tissue**	VAT
**Subcutaneous adipose tissue**	SAT
**Mass** Mass indicates the amount of the assessed tissue, corrected for the patient’s height. Higher scores indicate a higher volume of tissue	-M
**Radiation attenuation** Radiation attenuation indicates how much radiation is absorbed in tissues upon making a CT scan (expressed in Hounsfield units). Higher values indicate lower triglyceride concentration. For muscle, this indicates worse tissue quality. For fat, this indicates better nutritional reserves	-RA
**Short Nutritional Assessment Questionnaire** Higher scores indicate more malnutrition	SNAQ
**Groningen Frailty Index** Higher scores indicate more frailty	GFI

Additionally, these 3 tissues were assessed for radiation attenuation (RA). RA indicates how much radiation is absorbed in the body tissues (expressed in HU) during the diagnostic CT scan. The remaining radiation passes through the body and produces a grayscale image on CT. The RA of fat lies between − 190 and − 30 HU; the RA of water is per definition 0 HU; and the RA of muscle lies between − 29 and 150 HU. Hence, a decreased RA in fat could be indicative of better nutritional status (higher triglyceride concentration, lower water concentration), whereas a decreased RA in muscle could be indicative of worse muscle quality due to myosteatosis (higher triglyceride concentration) or muscle edema (higher water concentration).^25,27–31^ The RA of the 3 tissues will be referred to as: SM-RA, VAT-RA, and SAT-RA (Table 1).

### Z-Score

In an effort to correct for the effects of sex and standardize the scores, SM-M, VAT-M, SAT-M, SM-RA, VAT-RA, and SAT-RA were expressed as *Z*-scores. The *Z*-score depicts how each patient’s standard deviation differs from the mean value of patients of the same sex.^32^ It is calculated by taking the measured value of each patient and subtracting the sex-specific mean and thereafter dividing by the sex-specific standard deviation.

### Outcome Measurements

The primary outcome was the association of the 6 body composition Z-scores (SM-M, VAT-M, SAT-M, SM-RA, VAT-RA, and SAT-RA) with the occurrence of a major postoperative complication. Secondary outcomes were the association of the SNAQ score^23^ and GFI^24^ with the occurrence of a major postoperative complication. Postoperative complications were defined according to the Esophagectomy Complications Consensus Group (ECCG) definitions and scored according to the Clavien-Dindo Classification, as previously described.^21,33,34^ A major postoperative complication was defined as a Clavien-Dindo grade ≥ 3b complication.

### Statistical Analysis

Statistical analysis was performed using R statistical computing version 3.6.1 (R Foundation for Statistical Computing, Vienna, Austria). As previously described, the primary analysis included patients who underwent preoperative chemotherapy followed by gastrectomy. Subgroup analyses included patients who underwent primary surgery. The *Z*-scores of the body composition parameters were used, as previously described. Gaussian distributed continuous data are presented as means with standard deviations and non-Gaussian distributed continuous data as medians with interquartile ranges. Univariable and multivariable Poisson regression with robust error variances were performed for the binary outcome major postoperative complication yes/no, producing relative risks according to the methods by Zou et al.^35,36^. The 6 body composition *Z*-scores (SM-M, VAT-M, SAT-M, SM-RA, VAT-RA, and SAT-RA), SNAQ score, and GFI were each tested in a separate multivariable model without correction from the other 6 body composition *Z*-scores, SNAQ score, and GFI. Relevant baseline and treatment characteristics were first tested univariably and added to the multivariable models only if the *p* value was 0.200 or smaller. This was done to prevent over-fitting of the models.

## Results

### Patient Characteristics

**Fig. 1:**
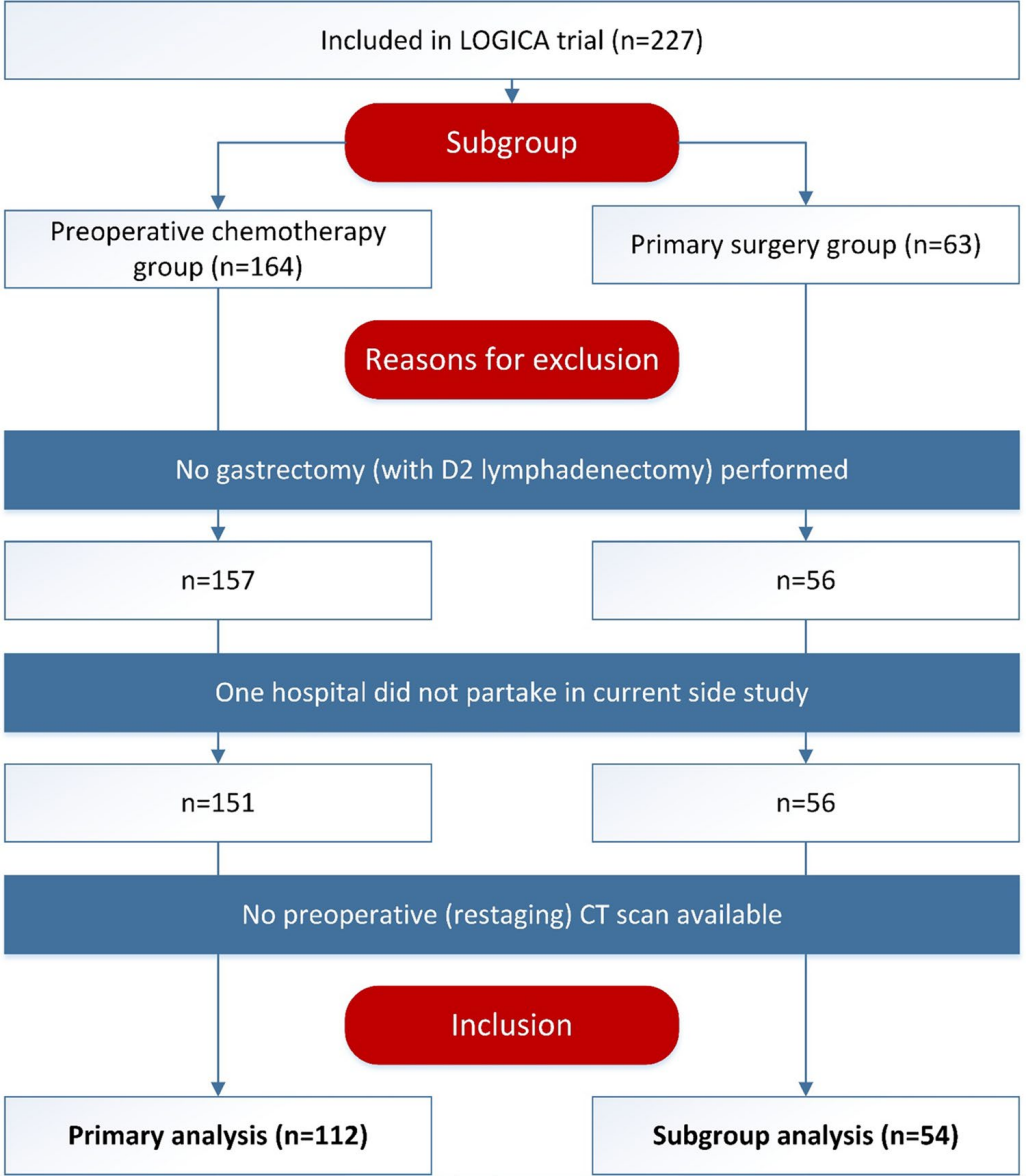
Study flowchart

From February 2015 to August 2018, 227 patients were included in the LOGICA trial in the 10 participating hospitals. A total of 164 patients received preoperative chemotherapy and 63 patients received primary surgery (Fig. 1).

Of the 164 patients in the preoperative chemotherapy group, 6 patients received a laparoscopy without resection, and 1 patient received an esophagogastric resection with cervical esophagostomy.^21^ The remaining 157 patients were potentially eligible for inclusion in the primary analysis. A total of 6 patients (4%) were excluded because one hospital chose not to partake in the current side study and 39 patients (25%) were excluded because no restaging CT scan was available. The remaining 112 patients (71%) were included in the primary analysis.

Of the 63 patients in the primary surgery group, 4 patients received a laparoscopy or laparotomy without resection, 1 patient received a distal gastrectomy with D1 lymphadenectomy, and 2 patients did not proceed to surgery.^21^ The remaining 56 were potentially eligible for inclusion in the subgroup analysis. After exclusion of 2 patients (4%) without available CT scans, the remaining 54 patients were included in the subgroup analysis.

Patient characteristics at baseline, body composition parameters, treatment characteristics, and outcomes are described in Table 2. In the preoperative chemotherapy group, preoperative chemotherapy was completed in 89 patients (79%) and stopped prematurely in 21 patients (19%), and data on completion were missing in two patients (2%). Total gastrectomy was performed in 50 patients (45%) and distal gastrectomy in 62 patients (55%). A grade ≥ 3b postoperative complication occurred in 9 patients (8%). The excluded 33 patients without a restaging CT scan had similar patient characteristics, treatment, and outcome as the included 112 patients (Supplementary Table 1).

**Table 2:** Patient characteristics, treatment and outcomes

	Preoperative chemotherapy		Primary surgery	
**n (%)**	112		54	
**Male sex**	73	(65.2)	32	(59.3)
**Age, years (mean (SD))**	65.6	(9.6)	74.7	(8.3)
**BMI, kg/m**^**2**^** (median [IQR])**	25.7	[23.2, 29.0]	25.4	[22.1, 28.1]
**ASA score**				
1	14	(12.5)	3	(5.6)
2	73	(65.2)	36	(66.7)
3	25	(22.3)	15	(27.8)
**Cardiovascular comorbidity**	55	(49.1)	38	(70.4)
**Pulmonary comorbidity**	23	(20.5)	12	(22.2)
**Location of tumor**				
Proximal stomach	14	(12.5)	3	(5.6)
Middle stomach	31	(27.7)	20	(37.0)
Distal stomach	67	(59.8)	31	(57.4)
**cT-stage**				
cT1	5	(4.5)	8	(14.8)
cT2	29	(25.9)	20	(37.0)
cT3	67	(59.8)	23	(42.6)
cT4	11	(9.8)	3	(5.6)
**cN1-3**	51	(45.5)	22	(40.7)
**Advanced cancer**^**1**^	88	(78.6)	32	(59.3)
**SNAQ score, (mean (SD))**	2	(2.1)	2.3	(2.5)
Missing	38	(34.9)	15	(27.8)
**GFI, (mean (SD))**	2.9	(2.3)	2.9	(2.3)
Missing	26	(23.2)	11	(20.4)
**SM, cm**^**2**^**/m**^**2**^** (mean (SD))**	44.8	(8.1)	42.8	(8.0)
**VAT, cm**^**2**^**/m**^**2**^** (mean (SD))**	51.9	(32.3)	57.5	(36.8)
**SAT, cm**^**2**^**/m**^**2**^** (mean (SD))**	63.8	(33.4)	58.6	(31.1)
**SM-RA, HU**^**2**^** (mean (SD))**	36.7	(10.7)	32.0	(8.0)
**VAT-RA, HU**^**2**^** (mean (SD))**	− 90.5	(7.8)	− 89.9	(8.8)
**SAT-RA, HU**^**2**^** (mean (SD))**	− 96.1	(8.9)	− 92.5	(9.7)
**Preoperative chemotherapy**				
ECC or equivalent	84	(75.0)	n/a	
FLOT	19	(17.0)	n/a	
Others	9	(8.0)	n/a	
**Preoperative chemotherapy completed (> 80% of courses)**				
Yes	89	(79.5)	n/a	
No	21	(18.8)	n/a	
Missing	2	(1.8)	n/a	
**Type of operation**				
Total gastrectomy	50	(44.6)	18	(33.3)
Distal gastrectomy	62	(55.4)	36	(66.7)
**Laparoscopic gastrectomy**	53	(47.3)	34	(63.0)
**Complication**	38	(33.9)	31	(57.4)
**CDC of most severe complication**	(%)			
1	8	(7.1)	2	(3.7)
2	16	(14.3)	12	(22.2)
3a	5	(4.5)	3	(5.6)
3b	2	( 1.8)	3	(5.6)
4a	4	(3.6)	2	(3.7)
4b	0	(0.0)	1	(1.9)
5	3	(2.7)	8	(14.8)
**Anastomotic leakage**	8	(7.1)	8	(14.8)
**Anastomotic leakage grade (ECCG)**				
I	2	(1.8)	1	(1.9)
II	1	(0.9)	0	(0.0)
III	5	(4.5)	7	(13.0)
**Adjuvant chemotherapy started**	59	(52.7)	1	(1.9)
**1-year all-cause mortality**	20	(17.9)	16	(29.6)

*IQR* interquartile range; *SD* standard deviation; *ASA* American Society of Anaesthesiologists; *SM* skeletal muscle; *SAT* subcutaneous adipose tissue; *VAT* visceral adipose tissue; *RA* radiation attenuation; *HU* Hounsfield units; *SNAQ* Short Nutritional Assessment Questionnaire; *GFI* Groningen Frailty Index. *ECC* epirubicin + cisplatin + capecitabine; *FLOT* fluorouracil + leucovorin + oxaliplatin + docetaxel; *CDC* Clavien-Dindo Classification; *ECCG* Esophagectomy Complications Consensus Group

^1^Defined as cT3-4N0 or cT1-2 N +

In the primary surgery group, total gastrectomy was performed in 18 patients (33%) and distal gastrectomy in 36 patients (67%). A grade ≥ 3b postoperative complication occurred in 14 patients (26%).

### CT Scan Timing

**Fig. 2:**
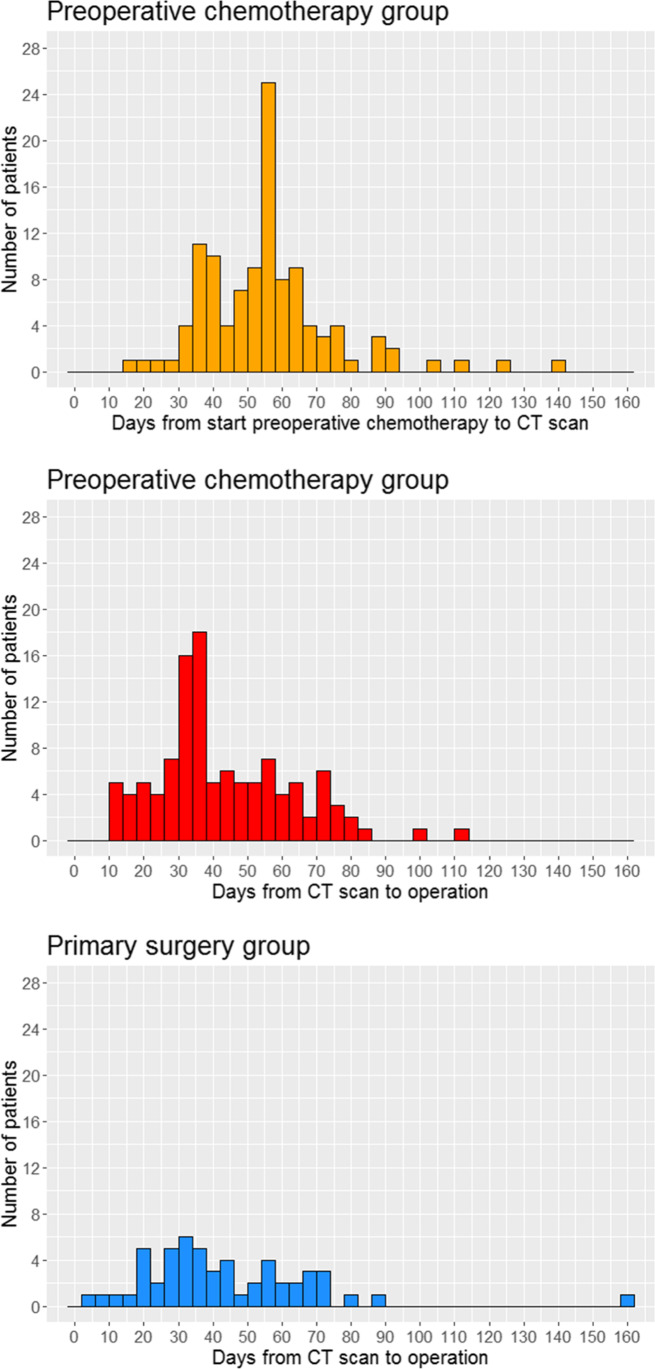
Histograms showing the timing of the CT scans. ^*^The primary surgery group has one outlier at 160 days. This patient underwent a staging CT scan, followed by an endoscopic submucosal dissection for early stage gastric cancer. Pathological analysis showed dubious radicality and angioinvasion, which prompted extensive cardiac screening of the patient due to comorbidity, followed by distal gastrectomy. This patient did not suffer a severe postoperative complication and was discharged in good clinical condition 10 days after surgery

The CT scan timing is displayed in Fig. 2. For the preoperative chemotherapy group, median time from start of preoperative chemotherapy to restaging CT scan was 56 days [IQR 42–63]. Median time from restaging CT scan to surgery was 37 days [IQR 31–55]. For the primary surgery group, median time from CT scan to surgery was 39 days [IQR 28–56].

## Primary Analyses: Preoperative Chemotherapy Group

### Tissue Mass

**Fig. 3:**
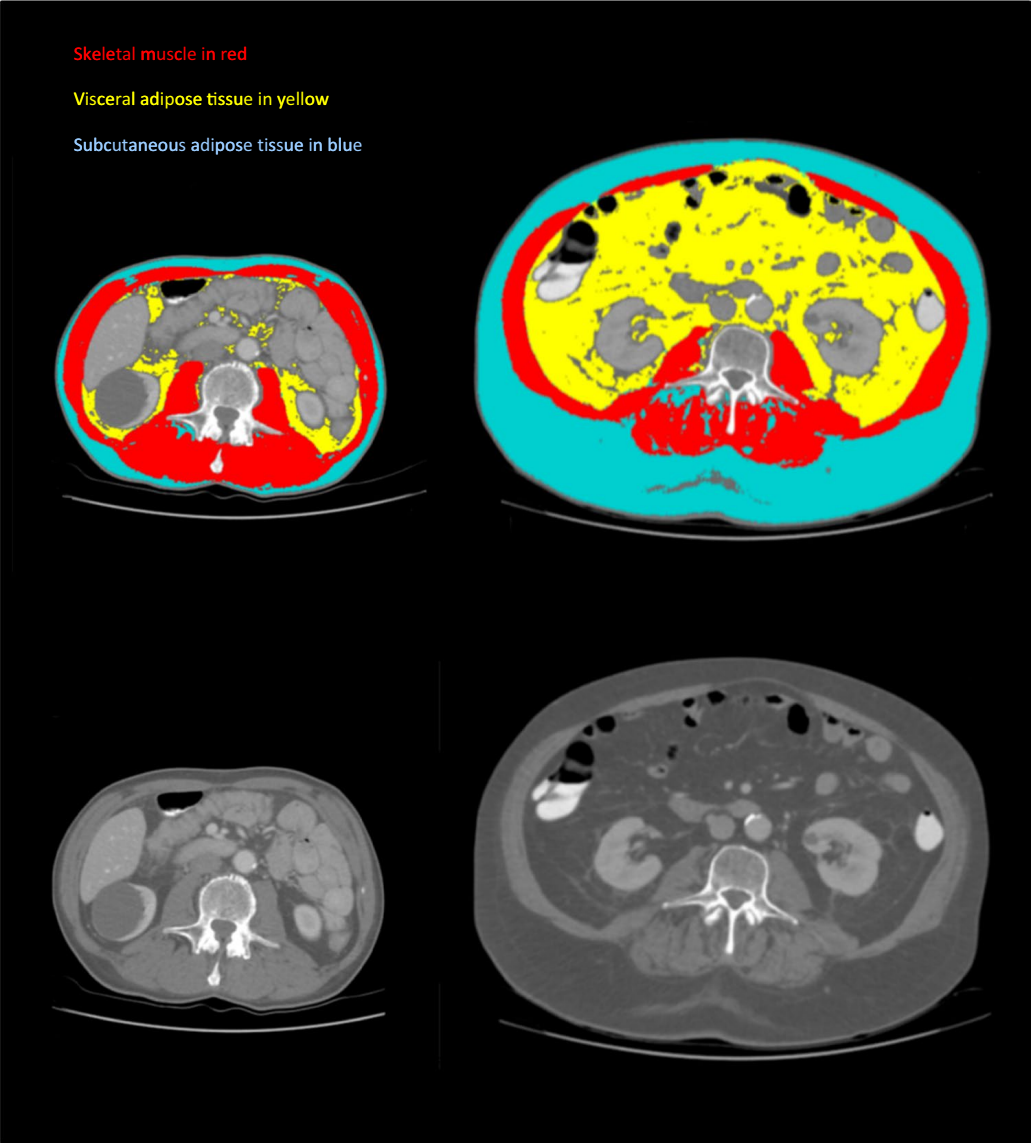
Example CT scans. In the top 2 scans SM, VAT, and SAT are delineated in red, yellow, and blue, respectively. The bottom 2 scans are the same scans without delineations. On the left, a patient is displayed with low *Z*-scores for VAT-M/SAT-M (low amount of fat) and high *Z*-scores for VAT-RA/SAT-RA (lighter shade of gray, indicative of low triglyceride concentration) On the right, a patient is displayed with high Z-scores for VAT-M/SAT-M (high amount of fat) and low *Z*-scores for VAT-RA/SAT-RA (darker shade of gray, indicative of high triglyceride concentration). The body composition of the patient on the left is associated with a higher rate of severe postoperative complications

In the preoperative chemotherapy group, a high SM-M *Z*-score (more muscle) was significantly associated with a lower risk of a grade ≥ 3b postoperative complication in univariable (RR 0.48, 95% CI 0.30–0.77, *p* = 0.002) and multivariable analyses (RR 0.47, 95% CI 0.28–0.78, *p* = 0.004) (Table 3, Fig. 3).

**Table 3:** Preoperative chemotherapy group. Relative risks of having a postoperative grade ≥ 3b complication

	Preoperative chemotherapy group						
	Univariable			Multivariable			
	RR	[95% CI]	*p*	RR	[95% CI]	*p*	
**SM-M *Z*-score**	0.48	[0.30–0.77]	**0.002**	0.47	[0.28–0.78]	**0.004**	*
**VAT-M *Z*-score**	0.47	[0.16–1.36]	0.164	0.44	[0.14–1.40]	0.166	*
**SAT-M *Z*-score**	0.64	[0.37–1.10]	0.105	0.61	[0.35–1.08]	0.088	*
**SM-RA *Z*-score**	0.95	[0.61–1.48]	0.821	0.95	[0.58–1.55]	0.825	*
**VAT-RA *Z*-score**	2.62	[1.39–4.94]	**0.003**	2.82	[1.52–5.23]	**0.001**	*
**SAT-RA *Z*-score**	2.00	[1.13–3.53]	**0.017**	1.95	[1.14–3.34]	**0.015**	*
**SNAQ score**	0.99	[0.70–1.42]	0.971	1.07	[0.79–1.44]	0.684	*
**GFI**	0.76	[0.52–1.11]	0.157	0.78	[0.56–1.10]	0.156	*
**Additional year of age**	1.00	[0.95–1.05]	0.980				
**ASA score**							
1 or 2	Ref	-	-				
3	0.99	[0.22–4.5]	0.994				
**cT stage**							
T1–T2	Ref	-	-				
T3–T4	1.53	[0.33–7.0]	0.586				
**cN stage**							
cN0	Ref	-	-				
cN1–cN3	0.96	[0.27–3.38]	0.945				
**Distal gastrectomy**	0.40	[0.11–1.53]	0.182	0.40	[0.10–1.58]	0.191	**
**Laparoscopic surgery**	0.89	[0.25–3.14]	0.857				

Poisson regressions with robust error variances were performed, producing a relative risk of having a postoperative grade ≥ 3b complication (yes/no) for each of the CT body composition parameters. Bold values indicate significance (*p* < 0.05)

*RR* relative risk; *CI* confidence interval; *ref* reference; *SM* skeletal muscle; *VAT* visceral adipose tissue; *SAT* subcutaneous adipose tissue; *M* mass; *RA* radiation attenuation; *SNAQ* Short Nutritional Assessment Questionnaire; *GFI* Groningen Frailty Index

^*^In multivariable analyses, each CT body composition parameter, the SNAQ score and GFI, was adjusted only for whether a total or distal gastrectomy was performed

^**^The displayed values for the variable distal gastrectomy are from the multivariable analysis in which SM-M *Z*-score and distal gastrectomy were included only. The values for the variable distal gastrectomy in the multivariable analyses of the remaining 5 CT body composition parameters, SNAQ score and GFI, were comparable (data not shown)

A high VAT-M *Z*-score (more visceral fat) showed a trend towards being associated with a lower risk of a grade ≥ 3b postoperative complication in univariable (RR 0.47, 95% CI 0.16–1.36, *p* = 0.164) and multivariable analyses (RR 0.44, 95% CI 0.14–1.40, *p* = 0.166), but did not reach statistical significance (Table 3).

Likewise, a high SAT-M *Z*-score (more subcutaneous fat) showed a trend towards being associated with a lower risk of a grade ≥ 3b postoperative complication in univariable (RR 0.64, 95% CI 0.37–1.10, *p* = 0.105) and multivariable analyses (RR 0.61, 95% CI 0.35–1.08, *p* = 0.088), but did not reach statistical significance (Table 3).

### Radiation Attenuation

In the preoperative chemotherapy group, a high SM-RA *Z*-score (good muscle quality) was not associated with a lower risk of a grade ≥ 3b postoperative complication in univariable (RR 0.95, 95% CI 0.61–1.48, *p* = 0.821) and multivariable analyses (RR 0.95, 95% CI 0.58–1.55, *p* = 0.825) (Table 3).

In contrast, a high VAT-RA *Z*-score (visceral fat depleted of triglycerides) was associated with a higher risk of a grade ≥ 3b postoperative complication in both univariable (RR 2.62, 95% CI 1.39–4.94, *p* = 0.003) and multivariable analyses (RR 2.82, 95% CI 1.52–5.23, *p* = 0.001) (Table 3).

Likewise, a high SAT-RA *Z*-score (subcutaneous fat depleted of triglycerides) was associated with a higher risk of a grade ≥ 3b postoperative complication in both univariable (RR 2.00, 95% CI 1.13–3.53, *p* = 0.017) and multivariable analyses (RR 1.95, 95% CI 1.14–3.34, *p* = 0.015) (Table 3).

### SNAQ and GFI

In the preoperative chemotherapy group, a high SNAQ score (more malnutrition) was not associated with an increased risk of a grade ≥ 3b postoperative complication in both univariable (RR 0.99, 95% CI 0.70–1.42, *p* = 0.971*)* and multivariable analyses (RR 1.07, 95% CI 0.79–1.44, *p* = 0.684) (Table 3). Likewise, a high GFI (more frailty) showed a trend towards being associated with a lower risk of a grade ≥ 3b postoperative complication in univariable (RR 0.76, 95% CI 0.52–1.11, *p* = 0.157) and multivariable analyses (RR 0.78, 95% CI 0.56–1.10, *p* = 0.156*)*, but did not reach statistical significance (Table 3).

### Total Versus Distal Gastrectomy

In the preoperative chemotherapy group, distal gastrectomy showed a trend towards being associated with a lower risk of a grade ≥ 3b postoperative complication in univariable analysis (RR 0.40, 95% CI 0.11–1.53, *p* = 0.182). Hence, in multivariable analyses, each CT body composition parameter, the SNAQ score and GFI, was adjusted for whether a total or distal gastrectomy was performed (Table 3).

## Subgroup Analysis: Primary Surgery Group

### Tissue Mass

In the primary surgery group, a high SM-M *Z*-score (more muscle) was significantly associated with an increased risk of a grade ≥ 3b postoperative complication in univariable (RR 1.47, 95% CI 1.22–1.77, *p* < 0.001) and multivariable analyses (RR 1.58, 95% CI 1.28–1.94, *p* < 0.001) (Table 4).

**Table 4:** Primary surgery group. Relative risks of having a postoperative grade ≥ 3b complication

	Primary surgery group						
	Univariable			Multivariable			
	RR	[95% CI]	*p*	RR	[95% CI]	*p*	
**SM-M *Z*-score**	1.47	[1.22–1.77]	** < 0.001**	1.58	[1.28–1.94]	** < 0.001**	*
**VAT-M *Z*-score**	1.06	[0.69–1.61]	0.798	1.17	[0.76–1.80]	0.466	*
**SAT-M *Z*-score**	0.96	[0.57–1.63]	0.883	1.04	[0.61–1.79]	0.875	*
**SM-RA *Z*-score**	1.50	[0.91–2.48]	0.109	0.59	[0.23–1.52]	0.277	*
**VAT-RA *Z*-score**	1.30	[0.91–1.85]	0.145	1.25	[0.85–1.83]	0.251	*
**SAT-RA *Z*-score**	1.23	[0.88–1.72]	0.221	1.25	[0.90–1.74]	0.178	*
**SNAQ score**	1.03	[0.86–1.24]	0.711	1.01	[0.85–1.20]	0.937	*
**GFI**	1.30	[1.17–1.45]	** < 0.001**	1.30	[1.16–1.45]	** < 0.001**	*
**Additional year of age**	0.99	[0.95–1.04]	0.810				
**ASA score**							
1 or 2	Ref	-	-				
3	1.44	[0.58–3.6]	0.432				
**cT stage**							
T1–T2	Ref	-	-				
T3–T4	1.44	[0.58–3.6]	0.438				
**cN stage**							
cN0	Ref	-	-				
cN1–cN3	0.81	[0.31–2.09]	0.660				
**Distal gastrectomy**	0.50	[0.21–1.21]	0.123	0.44	[0.18–1.06]	0.069	**
**Laparoscopic surgery**	0.59	[0.24–1.43]	0.243				

Poisson regressions with robust error variances were performed, producing a relative risk of having a postoperative grade ≥ 3b complication (yes/no) for each of the CT body composition parameters. Bold values indicate significance (*p* < 0.05)

*RR* relative risk; *CI* confidence interval; *ref* reference; *SM* skeletal muscle; *VAT* visceral adipose tissue; *SAT* subcutaneous adipose tissue; *M* mass; *RA* radiation attenuation; *SNAQ* Short Nutritional Assessment Questionnaire; *GFI* Groningen Frailty Index

^*^In multivariable analyses, each CT body composition parameter, the SNAQ score and GFI, was adjusted only for whether a total or distal gastrectomy was performed

^**^The displayed values for the variable distal gastrectomy are from the multivariable analysis in which SM-M *Z*-score and distal gastrectomy were included only. The values for the variable distal gastrectomy in the multivariable analyses of the remaining 5 CT body composition parameters, SNAQ score and GFI, were comparable (data not shown)

VAT-M and SAT-M (amount of fat) were not significantly associated with the occurrence of a grade ≥ 3b postoperative complication (Table 4).

### Radiation Attenuation

In the primary surgery group, SM-RA, VAT-RA, and SAT-RA (quality of muscle or fat) were not significantly associated with the occurrence of a grade ≥ 3b postoperative complication (Table 4).

### SNAQ and GFI

In the primary surgery group, a high SNAQ score (more malnutrition) was not associated with an increased risk of a grade ≥ 3b postoperative complication in univariable and multivariable analyses (Table 4). However, a high GFI (more frailty) was significantly associated with an increased risk of a grade ≥ 3b postoperative complication in univariable (RR per extra point 1.30, 95% CI 1.17–1.45, *p* < 0.001) and multivariable analyses (RR per extra point 1.30, 95% CI 1.16–1.45, *p* < 0.001) (Table 4).

### Total Versus Distal Gastrectomy

In the primary surgery group, distal gastrectomy showed a trend towards being associated with a lower risk of a grade ≥ 3b postoperative complication in univariable analysis (RR 0.50, 95% CI 0.21–1.21, *p* = 0.123). Hence, in multivariable analyses, each CT body composition parameter, the SNAQ score and GFI, was adjusted for whether a total or distal gastrectomy was performed (Table 4). 

## Discussion

This prospective multicenter study found that patients with a low skeletal muscle mass on preoperative restaging CT scan had a significantly higher risk of developing a major postoperative complication after preoperative chemotherapy followed by gastrectomy. Furthermore, patients with higher visceral or subcutaneous adipose tissue radiation attenuation (fat depleted of triglycerides) also had a significantly higher risk of developing a major postoperative complication. This is the first prospective multicenter study on the effects of body composition on postoperative complications in a Western population with predominantly advanced gastric cancer. These findings may help in better preoperative identification of high-risk patients.

A recent meta-analysis of Borggreve et al.^9^ concluded that patients with low skeletal muscle mass had an increased chance of developing (major) postoperative complications. However, only four retrospective studies from a Western population were included in this meta-analysis.^7,8,37,38^ Three studies (*n* = 36, *n* = 56, and *n* = 138) found a statistically significant association between sarcopenia and an increased risk of postoperative complications,^7,37,38^ whereas the study by Tegels et al. (*n* = 152) did not.^8^ The Tegels et al. study results might be explained due to the retrospective single-center design, introducing possible selection and historical bias. In addition, patients were likely in a poor condition since only 46.3% received preoperative chemotherapy, which is recommended for all eligible patients in the Western advanced gastric cancer population since 2006.^8^ Lastly, binary cut-off values for sarcopenia were used from the Prado et al. study, which were based on obese Canadian patients and were not externally validated.^12,13^ The current study does not have these limitations, due to the prospective design and the fact that all body composition parameters were expressed as continuous *Z*-scores.

Low skeletal muscle radiation attenuation (SM-RA) indicates a greater accumulation of lipids/fat in and around myocytes; this is called myosteatosis.^39^ The current study found no association between low skeletal muscle radiation attenuation (SM-RA) and postoperative complications after preoperative chemotherapy and gastrectomy. Literature on SM-RA in other abdominal cancers is ambiguous, with some studies demonstrating an association between low SM-RA and poor prognoses (possibly due to poor physical fitness),^11,16,31,40^ whereas other studies do not.^17,41–43^

Lower visceral adipose tissue radiation attenuation (VAT-RA) and subcutaneous adipose tissue radiation attenuation (SAT-RA) indicate a higher concentration of lipids/fat in the adipose tissue.^30^

This is a fairly new but very relevant outcome, which has been shown to be associated with worse outcomes in other abdominal cancers.^17,44^ In the current study on gastric cancer, low VAT-RA and SAT-RA (fat with high triglyceride concentration) were associated with a lower risk of developing a major postoperative complication after preoperative chemotherapy followed by gastrectomy. This effect might be due to the better nutritional status of these patients and increased lipid reserves. Whether this finding of low VAT-RA and SAT-RA on CT scan can also be seen intraoperatively, for example, as fat that easily tears was not investigated in the current study, but might be of interest for further research (Fig. 3). Of note, VAT-M and SAT-M (quantity of fat) were previously reported to be associated with VAT-RA and SAT-RA, indicating that all these variables are indicators of the patients’ lipid reserves. Indeed, VAT-M and SAT-M also showed a trend towards an association with major postoperative complications in the current study. Surprisingly, age and American Society of Anaesthesiologists (ASA) score were not significantly associated with the risk of developing a major postoperative complication in the current trial cohort, underlining the added value of the CT body composition parameters in predicting postoperative complications. Furthermore, the main LOGICA paper from which this manuscript is a side study of showed no difference between laparoscopic and open gastrectomy with respect to postoperative complications (44% vs 42%, *p* = 0.91).^21^ Moreover, both the laparoscopic and open study arm had comparable amount of patients who received total and distal gastrectomy, as the randomization was stratified for total/distal gastrectomy.

Importantly, since postoperative complications are associated with lower survival rates after gastroesophageal surgery, reducing postoperative complications is key.^45^ Nevertheless, it lies beyond the scope of the current study to determine whether the effect of body composition is prognostic and can’t be influenced (i.e., patients with a poor prognosis have poor preoperative body composition) or whether this effect can be influenced with therapeutic interventions (i.e., patients have poor postoperative outcomes due to poor preoperative body composition, improving body composition would improve outcomes).

Hence, based upon the current study data, the authors are not able to recommend whether additional nutritional replacement based on preoperative body composition is of additive value or not.

For patients undergoing primary surgery in the current study (*n* = 54), a lower skeletal muscle (SM) *Z*-score was associated with a significantly lower risk of developing a major postoperative complication. Strikingly, an opposite effect was found in the preoperative chemotherapy followed by gastrectomy group (*n* = 112). The primary surgery group result is presumably due to (selection) bias, though an actual effect cannot be excluded based upon the current study data. The authors believe results from the primary surgery group should be regarded with caution, since the primary surgery group is deemed to be representative of a more heterogeneous cohort of patients, who are in worse clinical condition and have a worse prognosis, compared to the more homogenous preoperative chemotherapy group. Indeed, the primary surgery group has a higher mean age (75 versus 66 years), higher prevalence of cardiovascular comorbidity (70% versus 49%), higher number of distal gastrectomies performed (67% versus 55%), higher occurrence of a grade ≥ 3b postoperative complication (26% versus 8%), and higher occurrence of 1-year mortality (30% versus 18%). The majority of patients in the primary surgery group were older, had advanced cancer, and, according to Dutch guidelines, should receive perioperative chemotherapy if eligible.^2,46^ Hence, a proportion of the primary surgery group was likely in poor clinical condition, deeming them not eligible for preoperative chemotherapy. The current study results underline that future research should analyze patients undergoing preoperative chemotherapy followed by surgery and patients undergoing the primary surgery as separate groups.

A higher frailty, indicated by the Groningen frailty index, showed a trend but no statistically significant association for the development of a major postoperative complication in the preoperative chemotherapy group.^47^ In the primary surgery group, a higher GFI (more frailty) was significantly associated with reduced occurrence of a major postoperative complication. As mentioned in the previous paragraph, results from the primary surgery group should be regarded with caution due to possible (selection) bias. Furthermore, a higher SNAQ score was not predictive for the development of a major postoperative complication in both the preoperative chemotherapy and the primary surgery group. The SNAQ was originally designed as a hospital screening tool for malnutrition.

Current literature highlights the effects of gastric cancer in relations to malnutrition and the development of cancer cachexia. Malnutrition could occur through physical obstruction of the gastrointestinal tract or systemic inflammation due to cancer.^43,48^ In the current trial, malnourished patients’ nutrition was preoperatively optimized according to standard care, based upon the national guidelines and the guidelines of Enhanced Recovery After Surgery (ERAS).^22,49^ Perhaps no association was found between the SNAQ score and major postoperative complications, due to the SNAQ being a subjective patient reported outcome, which was not specifically designed for scientific purposes in a trial cohort. The authors believe CT body composition measures are more objective and thus more reliable.

In the preoperative chemotherapy group, the restaging CT scans, and not the initial staging CT scans, were used to determine the patients’ body composition. The restaging CT scans were expected to give the best uniform representation of the patients’ condition during surgery, since body composition often changes during preoperative chemotherapy.^19,50–52^

A limitation of the current study is the exclusion of 39 patients, due to the unavailability of a restaging CT scan. Since the current study was an observational prospective side study of the LOGICA trial, the restaging CT scan was not obligatory but made according to standard of care. Even though the multidisciplinary tumor board decided when a restaging CT scan did not have to be made, these missings appear to have occurred at random, since patient characteristics, treatment, and outcome did not change upon including these 39 patients (Online Resource 1). Thus, selection bias is presumably limited. It is considered a strength of the current study that the timing of the CT scans was reported in detail, which is not the case in the majority of studies in the recent Borggreve et al. meta-analysis.^9^

In addition, the average BMI of our cohort was relatively low (~ 25) when compared to that of the American, Canadian, or South American population. This is representative for the typical West-European population with gastric cancer. Considering this, one could argue that the findings of this study cannot necessarily be extrapolated to populations with a higher BMI.^3^ The occurrence of any postoperative complication is used as an outcome in some body composition studies in literature, whereas other studies use only postoperative complications of a certain Clavien-Dindo grade.^9^ In the current study, a grade ≥ 3b complication was used, since predicting this grade preoperatively is deemed to be the most useful to guide clinical decision making. In the preoperative chemotherapy group, the point estimated relative risks for the SM-M, VAT-RA, and SAT-RA *Z*-scores were 0.47, 2.82, and 1.95, respectively. Hence, a patient with a VAT-RA of 1 standard deviation above the study population mean (belonging to the 16% highest VAT-RA values in the study population) would have almost 3 times the estimated chance of developing a grade ≥ 3b postoperative complication, compared to the patient with an average VAT-RA (Fig. 3).

Based upon the current study results, routine assessment and collection of CT body composition could be implemented in standard oncological care of gastric cancer patients. Once large prospectively collected datasets with continuous variables for CT body composition, known predictors such as age, ASA grade, and type of resection and postoperative complication rates, are available to serve as population reference values, body composition can be used to guide clinical decision making for the individual patient.^53^ Body composition analysis could then be used during preoperative multidisciplinary tumor board discussions to objectively and reproducibly predict the relative risk of a major postoperative complication.

In conclusion, this prospective multicenter study demonstrated that low skeletal muscle mass and a high visceral or subcutaneous adipose tissue radiation attenuation (fat depleted of triglycerides) are strong predictors of developing a major postoperative complication in gastric cancer patients treated with preoperative chemotherapy followed by gastrectomy. Incorporating body composition analysis could lead to a better selection of at-risk patients for major postoperative complications and aid in treatment decision-making.

## Collaborators (LOGICA study group)

Juul J.W. Tegels^1^, Karel W.E. Hulsewe^1^, Hylke J.F. Brenkman^2^, Maarten F.J. Seesing^2^, Grard A.P. Nieuwenhuijzen^3^, Jeroen EH Ponten^3^, Bas P.L. Wijnhoven^4^, Sjoerd M Lagarde^4^, Wobbe O de Steur^5^, Henk H Hartgrink^5^, Ewout A Kouwenhoven^6^, Marc J van Det^6^, Eelco B Wassenaar^7^, Edwin S van der Zaag^7^, Werner A Draaisma^8^, Ivo A.M.J. Broeders^8^, Suzanne S Gisbertz^9^, Mark I van Berge Henegouwen^9^, Hanneke W. M. van Laarhoven^10^.

## Supplementary Information

Below is the link to the electronic supplementary material.

Supplementary file Table 1 (DOCX 21 KB)
