# Supplementary material for: Body Composition Is a Predictor for Postoperative Complications After Gastrectomy for Gastric Cancer: a Prospective Side Study of the LOGICA Trial
Source: J Gastrointest Surg. 2022 Apr 29;26(7):1373–87. doi: 10.1007/s11605-022-05321-0 (PMC9296433; doi:10.1007/s11605-022-05321-0)
Supplement: Supplementary file 1 — Supplementary file Table 1 (DOCX 21 KB) [file 11605_2022_5321_MOESM1_ESM.docx]

**Supplementary Table 1** patient characteristics, treatment and outcome of the preoperative chemotherapy group with and without exclusion of patients with no available restaging CT-scan.

|  | Preoperative chemotherapy, main study population | | | Preoperative chemotherapy, patients without restaging CT-scan not excluded | |
| --- | --- | --- | --- | --- | --- |
| **n (%)** | 112 |  |  | 151 |  |
| **Male sex** | 73 | ( 65.2) |  | 98 | ( 64.9) |
| **Age, years (mean (SD))** | 65.6 | ( 9.6) |  | 65.1 | (10.1) |
| **BMI, kg/m^2^ (median [IQR])** | 25.7 | [23.2, 29.0] |  | 25.2 | [22.7, 28.6] |
| **ASA score** |  |  |  |  |  |
| 1 | 14 | ( 12.5) |  | 17 | ( 11.3) |
| 2 | 73 | ( 65.2) |  | 99 | ( 65.6) |
| 3 | 25 | ( 22.3) |  | 35 | ( 23.2) |
| **Cardiovascular comorbidity** | 55 | ( 49.1) |  | 77 | ( 51.0) |
| **Pulmonary comorbidity** | 23 | ( 20.5) |  | 31 | ( 20.5) |
| **Location of tumor** |  |  |  |  |  |
| Proximal stomach | 14 | ( 12.5) |  | 22 | ( 14.6) |
| Middle stomach | 31 | ( 27.7) |  | 44 | ( 29.1) |
| Distal stomach | 67 | ( 59.8) |  | 85 | ( 56.3) |
| **cT-stage** |  |  |  |  |  |
| cT1 | 5 | ( 4.5) |  | 6 | ( 4.0) |
| cT2 | 29 | ( 25.9) |  | 41 | ( 27.2) |
| cT3 | 67 | ( 59.8) |  | 91 | ( 60.3) |
| cT4 | 11 | ( 9.8) |  | 13 | ( 8.6) |
| **cN1-3** | 51 | ( 45.5) |  | 70 | ( 46.4) |
| **Advanced cancer^1^** | 88 | ( 78.6) |  | 121 | ( 80.1) |
| **SNAQ score, (mean (SD))** | 2 | ( 2.05) |  | 2.18 | (2.09) |
| Missing | 38 | ( 34.9) |  | 48 | ( 31.8) |
| **GFI, (mean (SD))** | 2.9 | ( 2.3) |  | 3.08 | (2.34) |
| Missing | 26 | (23.2) |  | 37 | ( 24.5) |
| **Preoperative chemotherapy** |  |  |  |  |  |
| ECC or equivalent | 84 | ( 75.0) |  | 114 | ( 75.5) |
| FLOT | 19 | ( 17.0) |  | 26 | ( 17.2) |
| Other | 9 | ( 8.0) |  | 11 | ( 7.3) |
| **Preoperative chemotherapy completed (>80% of courses)** |  |  |  |  |  |
| Yes | 89 | ( 79.5) |  | 120 | ( 79.5) |
| No | 21 | ( 18.8) |  | 29 | ( 19.2) |
| Missing | 2 | ( 1.8) |  | 2 | ( 1.3) |
| **Type of operation** |  |  |  |  |  |
| Total gastrectomy | 50 | ( 44.6) |  | 69 | ( 45.7) |
| Distal gastrectomy | 62 | ( 55.4) |  | 82 | ( 54.3) |
| **Laparoscopic gastrectomy** | 53 | ( 47.3) |  | 69 | ( 45.7) |
| **Complication** | 38 | ( 33.9) |  | 58 | ( 38.4) |
| **CDC of most severe complication** | |  |  |  |  |
| 1 | 8 | ( 7.1) |  | 10 | ( 6.6) |
| 2 | 16 | ( 14.3) |  | 24 | ( 15.9) |
| 3a | 5 | ( 4.5) |  | 6 | ( 4.0) |
| 3b | 2 | ( 1.8) |  | 7 | ( 4.6) |
| 4a | 4 | ( 3.6) |  | 6 | ( 4.0) |
| 4b | 0 | ( 0.0) |  | 0 | ( 0.0) |
| 5 | 3 | ( 2.7) |  | 5 | ( 3.3) |
| **Anastomotic leakage** | 8 | ( 7.1) |  | 12 | ( 7.9) |
| **Anastomotic leakage grade (ECCG)** | |  |  |  |  |
| I | 2 | ( 1.8) |  | 2 | ( 1.3) |
| II | 1 | ( 0.9) |  | 2 | ( 1.3) |
| III | 5 | ( 4.5) |  | 8 | ( 5.3) |
| **Adjuvant chemotherapy started** | 59 | ( 52.7) |  | 79 | ( 52.3) |
| **1-year all-cause mortality** | 20 | ( 17.9) |  | 24 | ( 15.9) |
| **Supplementary Table 1** patient characteristics, treatment and outcome of the preoperative chemotherapy group with and without exclusion of patients with no available restaging CT-scan. IQR = interquartile range; SD = standard deviation; ASA = American Society of Anaesthesiologists; SM = skeletal muscle; SAT = subcutaneous adipose tissue; VAT = visceral adipose tissue; RA = radiation attenuation; HU = Hounsfield units; SNAQ = Short Nutritional Assessment Questionnaire; GFI = Groningen Frailty Index. ECC = epirubicin + cisplatin + capecitabine, FLOT = fluorouracil + leucovorin + oxaliplatin + docetaxel , CDC = Clavien-Dindo Classification; ECCG = Esophagectomy Complications Consensus Group. ^1^Defined as cT3-4N0 or cT1-2N+. | | | | | |
